# Analysis of clinical outcomes and prognostic factors in patients treated with definitive chemoradiotherapy for oesophageal squamous cell carcinoma

**DOI:** 10.1002/cam4.3783

**Published:** 2021-02-15

**Authors:** Hyehyun Jeong, Hyeon‐Su Im, Yeonghak Bang, Yong‐Hee Kim, Hyeong Ryul Kim, Hyun Joo Lee, Hwoon‐Yong Jung, Gin Hyug Lee, Ho June Song, Do Hoon Kim, Kee Don Choi, Jeong Hoon Lee, Ji Yong Ahn, Hee Kyong Na, Jin‐Sook Ryu, Jihoon Kang, Sung‐Bae Kim, Jong Hoon Kim, Sook Ryun Park

**Affiliations:** ^1^ Department of Oncology Asan Medical Center University of Ulsan College of Medicine Seoul Republic of Korea; ^2^ Division of Hematology and Oncology Department of Internal Medicine Ulsan University Hospital University of Ulsan College of Medicine Ulsan Republic of Korea; ^3^ Department of Thoracic and Cardiovascular Surgery Asan Medical Center University of Ulsan College of Medicine Seoul Republic of Korea; ^4^ Department of Radiology Asan Medical Center University of Ulsan College of Medicine Seoul Republic of Korea; ^5^ Department of Gastroenterology Asan Medical Center University of Ulsan College of Medicine Seoul Republic of Korea; ^6^ Department of Nuclear Medicine Asan Medical Center University of Ulsan College of Medicine Seoul Republic of Korea; ^7^ Division of Hematology/Oncology Department of Internal Medicine Kangbuk Samsung Hospital Sungkyunkwan University School of Medicine Seoul Republic of Korea; ^8^ Department of Radiation Oncology Asan Medical Center University of Ulsan College of Medicine Seoul Republic of Korea

**Keywords:** clinical response, definitive chemoradiotherapy, oesophageal cancer, prognosis

## Abstract

As patients receiving definitive chemoradiotherapy (dCRT) for oesophageal squamous cell carcinoma (ESCC) are heterogeneous, we aimed to identify prognostic factors and failure patterns after dCRT. From 2006 to 2015, 327 patients who received dCRT for ESCC were reviewed. Treatment response to dCRT was evaluated based on EORTC‐PET criteria with endoscopy and CT results. After dCRT, 296 patients (90.5%) achieved disease stabilisation, with 132 cases of complete response (CR) (40.4%), 158 of partial response (PR) (48.3%) and 6 of stable disease (SD) (1.8%); 31 patients (9.5%) had progressive disease (PD). Median overall survival (OS) from response evaluation was 24.0 months in the overall population. Post‐treatment clinical response was the most significant prognostic factor for OS in the multivariate analysis (median OS, 65.0 months for CR, 17.3 months for PR, 4.4 months for SD and 4.0 months for PD; *p* < 0.0001). Median progression‐free survival (PFS) in 296 patients who achieved disease stabilisation was 13.1 months, and only clinical response was a significant factor in the multivariate analysis. The median PFS of CR, PR and SD patients were 36.9, 9.2 and 2.8 months, respectively (*p* < 0.0001). The clinical response was also significantly associated with the predominant failure pattern (locoregional failure [81.6%] in the initial non‐PD group vs. distant metastasis [87.1%] in the initial PD group [*p* < 0.0001]). In conclusion, definitive chemoradiotherapy‐treated ESCC patients showed highly different prognoses after treatment especially according to the clinical response to chemoradiotherapy.

## Graphical abstract

Patients with oesophageal squamous cell carcinoma treated with definitive chemoradiotherapy show highly different prognoses after treatment, especially according to the clinical response to chemoradiotherapy.

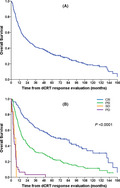

## 1 INTRODUCTION

Oesophageal cancer is the seventh most common cancer and the sixth most common cause of cancer death globally.^1^ Oesophageal cancer research is crucial because of the rapidly fatal course of the cancer and an advanced stage presentation.^2^, ^3^ Although surgical resection remains the gold standard of treatment for localised resectable oesophageal cancer, the addition of chemotherapy and radiotherapy has proven necessary for enhancing locoregional control and survival in patients with locally advanced disease.^4^, ^5^ Advances in surgical techniques and perioperative care have improved short‐term outcomes considerably by decreasing operative mortality; however, oesophagectomy is still associated with significant surgical mortality and morbidity rates. Definitive chemoradiotherapy is the standard therapy for locally advanced unresectable oesophageal cancer, as well as for patients who cannot tolerate or decline surgery. Several clinical studies have reported comparable survival outcomes between definitive chemoradiotherapy and preoperative chemoradiotherapy plus surgery, particularly in patients who respond to chemoradiation.^6^, ^7^ However, although definitive chemoradiotherapy results in favourable short‐term outcomes in most patients, with overall response rates ranging from 65% to 98%, persistent or recurrent disease is frequent after definitive chemoradiation.^8^, ^9^, ^10^ Despite these unsatisfactory treatment outcomes with definitive chemoradiotherapy, the current common clinical practice is surveillance without further treatment until disease progression or recurrence in patients who showed response or disease stabilisation after chemoradiotherapy. As patients receiving definitive chemoradiotherapy are heterogeneous in terms of potential prognostic factors, treatment strategies after definitive chemoradiotherapy need to be stratified based on different prognoses for these patients. Accordingly, the aims of the present study were to identify prognostic factors in locoregional oesophageal cancer patients treated with definitive chemoradiotherapy and to evaluate failure patterns after treatment.

## 2 METHODS

### 2.1 Patients

A total of 464 patients who received definitive chemoradiotherapy for localised oesophageal cancer from January 2006 to October 2015 at Asan Medical Center, a tertiary referral centre in Seoul, Republic of Korea, were retrospectively screened for study recruitment. All patients met the following inclusion criteria; (i) Age ≥18 years old; (ii) histologically confirmed oesophageal squamous cell carcinoma; (iii) clinical T1b or higher, any N, M0 (except for supraclavicular lymph node metastasis only as a M1 lesion, which was included) according to the eighth edition of the American Joint Committee on Cancer (AJCC) staging system^11^; (iv) unresectable disease, resectable but medically inoperable disease or patient's refusal of surgery; (v) total dose of radiotherapy as a component of definitive chemoradiotherapy ≥40 Gy; (vi) available clinical data for staging and assessing treatment response to definitive chemoradiotherapy using computed tomography (CT), oesophagogastroduodenoscopy (EGD), endoscopic ultrasound (EUS) and ^18^F‐fluorodeoxyglucose positron emission tomography (FDG‐PET); (vii) no sequential surgery right after the completion of chemoradiotherapy; and (viii) no other concurrent malignancy that might have affected clinical outcomes. A total of 137 patients were excluded due to a total radiation dose of under 40 Gy (*n* = 25), lack of PET data in the response evaluation after definitive chemoradiotherapy (*n* = 76) and other concurrent malignancy (*n* = 36), leaving 327 patients eligible for the analysis.

This study was approved by the Institutional Review Board of Asan Medical Center, and was performed in accordance with the ethical standards of the institutional research committee and the latest Declaration of Helsinki. IRB granted a waiver of informed consent for this study.

### 2.2 Treatments

Definitive chemoradiotherapy consisted of concurrently administered fluoropyrimidines plus platinum, including capecitabine/cisplatin, S‐1/oxaliplatin and 5‐fluorouracil/cisplatin or platinum ±taxane with radiotherapy. Radiotherapy was delivered once a day to a total dose of 40–62 Gy (typical total dose of 50.4 Gy) in 25–30 fractions of 1.8–2 Gy with a 15‐MV linear accelerator. The clinical target volume included the primary tumour with a 5‐cm craniocaudal margin and 2‐cm lateral margin and regional lymph nodes. Supraclavicular lymph nodes were routinely encompassed in upper thoracic oesophageal cancers and celiac lymph nodes in distal or middle thoracic oesophageal cancers. Before definitive chemoradiotherapy, one or two cycles of induction chemotherapy were administered according to institutional practice; however, induction chemotherapy was omitted in some cases at the discretion of the clinicians. Patients whose disease had progressed after completing definitive chemoradiotherapy received subsequent treatment at the discretion of the physician, whereas those who had achieved disease stabilisation including complete response (CR), partial response (PR) or stable disease (SD) were followed up without any anti‐cancer treatment until disease progression.

### 2.3 Staging and response evaluation

The initial staging work up included CT, EGD, EUS and FDG‐PET. CT, EGD and FDG‐PET were performed in all patients included in this study, and EUS could not be performed in 29 patients due to severe oesophageal obstruction. Tumour response assessment using CT, EGD and PET was performed 4–8 weeks after the end of definitive chemoradiotherapy; however, an evaluation schedule outside of this range was allowed according to the clinical situation. Endoscopic biopsy for suspicious lesions at the time of response evaluation was performed unless it was technically infeasible or was associated with unacceptable clinical risk. Metabolic response using PET was assessed based on European Organisation for Research and Treatment of Cancer (EORTC) criteria: CR, complete resolution of FDG uptake in all lesions; PR, greater than 25% reduction in the sum of SUVmax; progressive disease (PD), more than 25% increase in the sum of SUVmax or appearance of new FDG‐avid lesions; and SD, does not qualify for CR, PR or PD.^12^ Overall tumour response to definitive chemoradiotherapy was classified mainly based on EORTC‐PET criteria^12^ with the results of EGD and CT as follows:

- Clinical CR was defined as no radiographic, endoscopic or metabolic evidence of disease: (i) no residual tumour visible on EGD, which included no mucosal lesions such as erosion, a granular protruded lesion or ulceration except for only a flat white scar and no residual cancer cells in endoscopic biopsy. The lack of biopsy results alone did not exclude clinical CR if gross endoscopic findings were consistent with the above criteria; (ii) complete resolution of FDG uptake within all lesions, making them indistinguishable from the surrounding tissue^12^; and (iii) CR on CT according to Response Evaluation Criteria in Solid Tumours (RECIST) v1.1.^13^
- Clinical PR was defined as (i) PR according to EORTC‐PET criteria without evidence of disease progression on both EGD and CT (RECIST v1.1) or (ii) CR according to EORTC‐PET criteria without evidence of disease progression on CT (RECIST v1.1) but with evidence of disease on EGD.
- Clinical SD was defined as SD according to EORTC‐PET criteria without evidence of disease progression on both EGD and CT (RECIST v1.1).
- Clinical PD was defined as PD according to EORTC‐PET criteria, EGD or CT (RECIST v1.1).

In the assessment of the response at the primary tumour site, if there was distinguishable focal FDG uptake at the primary tumour site in the case of diffuse FDG uptake in the oesophagus suggesting radiotherapy‐induced oesophagitis, it was considered as non‐CR and classified according to EROTC‐PET criteria. However, if there was no focal FDG uptake at the primary tumour site distinguishable from the surrounding oesophagitis, the above endoscopic CR definition (in which mucosal lesions due to oesophagitis do not exclude CR) determined whether it was considered as CR or non‐CR (PR). In this study, overall tumour response was used for survival analyses.

### 2.4 Data collection and statistics

Clinical data were abstracted from the patients’ medical records, and tumour assessments were reviewed. The neutrophil‐to‐lymphocyte ratio (NLR) was defined as the absolute neutrophil count divided by the absolute lymphocyte count. The platelet‐to‐lymphocyte ratio (PLR) was defined as the absolute platelet count divided by the absolute lymphocyte count. The pre‐treatment NLR, PLR, serum albumin level and body weight were measured at the first day of treatment, and the above‐mentioned parameters at post‐treatment were measured at the time of post‐definitive chemoradiotherapy evaluation. Overall survival (OS) was measured from the date of definitive chemoradiotherapy response evaluation to death by any cause. Progression‐free survival (PFS) was measured from the date of definitive chemoradiotherapy response evaluation to disease progression or death of any cause, whichever occurred first. Baseline patient characteristics were assessed using a descriptive method. OS and PFS were estimated using the Kaplan–Meier method and compared using the log‐rank test. We carried out univariate and multivariate analyses of survival times using the Cox proportional hazards model. We chose age, sex, Eastern Cooperative Oncology Group (ECOG) performance status, tumour location, histologic grade of the tumour, clinical stage, the presence or absence of induction chemotherapy, overall response to definitive chemoradiotherapy, weight loss during chemoradiotherapy, pre‐ and post‐treatment albumin, PLR and NLR as potential prognostic variables. Multivariate analyses were carried out only for variables that has shown *p* values <0.05 in univariate Cox regression analyses. All tests were two‐sided and *p* values of <0.05 were considered statistically significant.

## 3 RESULTS

### 3.1 Patient characteristics

Table 1 shows the baseline characteristics of patients. More than half of patients (54.1%) had clinical stage III or IV disease with cT3–4 (61.5%) and cN1–3 (65.4%) tumours. The most common reason for receiving definitive chemoradiotherapy was the patient's refusal of surgery (40.1%) followed by unresectable disease (35.2%) and medical comorbidity (24.8%). Most commonly used radiotherapy method was three‐dimensional radiotherapy in 232 patients (70.9%), followed by two‐dimensional radiotherapy in 86 patients (26.3%) and intensity modulated radiation therapy in five patients (1.5%).

**TABLE 1:** Baseline characteristics (*n* = 327)

Characteristics		No. of patients
Age, years (range)	Median	66 (40–84)
Sex	Male	308 (94.2%)
ECOG performance status	0	67 (20.5%)
	1	203 (62.1%)
	2	11 (3.4%)
	Unknown	46 (14.1%)
Tumour location	Cervical (UI 15–20 cm)	12 (3.7%)
	Upper thoracic (UI 20–25 cm)	44 (13.5%)
	Mid thoracic (UI 25–30 cm)	106 (32.4%)
	Lower thoracic (UI 30–40 cm)	165 (50.5%)
Histologic grade	G1 (W/D)	46 (14.1%)
	G2 (M/D)	213 (65.1%)
	G3 (P/D)	44 (13.5%)
	GX (not assessed)	24 (7.3%)
Clinical T stage^a^	T1	42 (12.8%)
	T2	84 (25.7%)
	T3	166 (50.8%)
	T4	35 (10.7%)
Clinical N stage^a^	N0	113 (34.6%)
	N1	164 (50.2%)
	N2	48 (14.7%)
	N3	2 (0.6%)
Clinical TNM stage^a^	I	36 (11.0%)
	II	114 (34.9%)
	III	101 (30.9%)
	IVA	23 (7.0%)
	IVB (SCN metastasis only as M1)	53 (16.2%)
Reason for dCRT	Patient's refusal	131 (40.1%)
	Medical comorbidity	81 (24.8%)
	Unresectable disease	115 (35.2%)
Induction chemotherapy	Done	304 (93.0%)
	Not done	23 (7.0%)
Chemotherapy regimen for dCRT	Capecitabine and cisplatin	294 (89.9%)
	S−1 and oxaliplatin	22 (6.7%)
	5‐FU and cisplatin	6 (1.8%)
	Paclitaxel and cisplatin	3 (0.9%)
	Cisplatin or carboplatin only	2 (0.6%)
Total radiation dose (Gy)	Median	50.4 (40–62)
Weight change during dCRT	Median	−4.1% (−28.0% to +33.1%)
Serum albumin level (g/dL)	Pre‐treatment, median	3.8 (2.4–4.9)
	Post‐treatment, median	3.6 (1.8–4.8)
Neutrophil‐to‐lymphocyte ratio	Pre‐treatment, median	2.0 (0.1–28.9)
	Post‐treatment, median	2.2 (0.2–18.7)
Platelet‐to‐lymphocyte ratio	Pre‐treatment, median	119.4 (30.4–609.5)
	Post‐treatment, median	127.4 (6.8–780.6)

Data are the median (range) or number (%).

Abbreviations: 5‐FU, 5‐fluorouracil; dCRT, definitive chemoradiotherapy; ECOG PS, Eastern Cooperative Oncology Group performance status; M/D, moderately differentiated; P/D, poorly differentiated; SCN, supraclavicular node; UI, upper incision; W/D, well differentiated.

^a^ Clinical staging was done according to the eighth edition of the American Joint Committee on Cancer staging system.

### 3.2 Tumour response to definitive chemoradiotherapy

The evaluation of metabolic response to definitive chemoradiotherapy showed CR in 146 patients (44.6%), PR in 118 patients (36.1%), SD in 7 patients (2.1%) and PD in 27 patients (8.3%); however, 29 patients were not evaluable due to the presence of diffuse oesophagitis (*n* = 26, 8.0%) or non‐FDG‐avid tumours (*n* = 3, 0.9%) (Table 2). Overall treatment response was assessed by PET in combination with CT and EGD results, except for 20 patients whose endoscopic response could not be evaluated due to obstruction. PET and CT were performed in all patients. As a result, 132 patients (40.4%) showed CR, 158 patients (48.3%) showed PR, 6 patients (1.8%) had SD and 31 patients (9.5%) had PD (Table 2). Among 26 patients who showed diffuse oesophagitis on PET, 7 (26.9%) and 19 (73.1%) achieved clinical CR and clinical PR, respectively. In the 146 patients who achieved complete metabolic response, 23 (15.8%) had residual disease on EGD and classified into overall PR group. Among the 152 patients with non‐progressive metabolic disease, 4 (2.6%) was classified into PD group by CT or EGD response (Table S1).

**TABLE 2:** Tumour response to definitive chemoradiotherapy (*n* = 327)

Metabolic response according to EORTC‐PET criteria	No. of patients
Complete metabolic response	146 (44.6%)
Partial metabolic response	118 (36.1%)
Stable metabolic disease	7 (2.1%)
Progressive metabolic disease	27 (8.3%)
Diffuse oesophagitis	26 (8.0%)
Non‐FDG avid tumour	3 (0.9%)
Overall clinical response^a^	
Complete response	132 (40.4%)
Partial response	158 (48.3%)
Stable disease	6 (1.8%)
Progressive disease	31 (9.5%)

^a^ Overall response was classified mainly based on EORTC‐PET criteria^12^ with the results of endoscopy and computed tomography.

### 3.3 Survival outcomes and prognostic factor analysis

**FIGURE 1:**
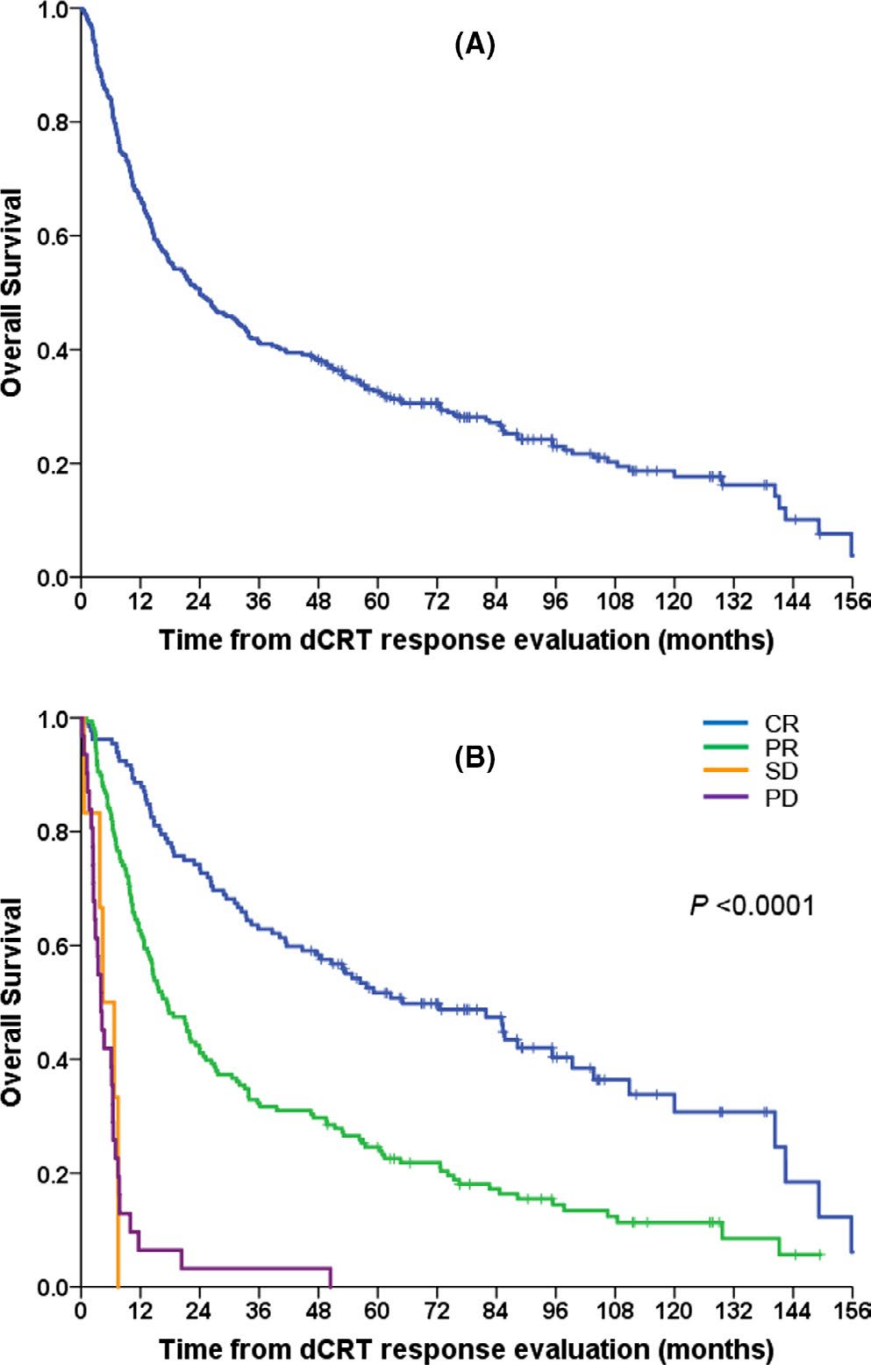
(A) Overall survival curve of the entire study population and (B) overall survival curves according to overall tumour response to definitive chemoradiotherapy (dCRT)

The median follow‐up duration from the date of post‐treatment evaluation was 95.4 months (range: 0.2–158.0 months). The median OS from post‐treatment response evaluation in the entire study population was 24.0 months (95% CI, 16.9–31.1) (Figure 1A). The survival rates at 1, 3 and 5 years from post‐treatment response evaluation were 66.7% (95% CI, 61.6–71.8), 41.3% (95% CI, 35.9–46.6) and 32.7% (95% CI, 27.6–37.8), respectively (Figure 1A). Univariate analysis for OS in the entire patient population showed that sex, ECOG performance status, clinical TNM stage, reason for definitive chemoradiotherapy, weight loss during chemoradiotherapy, overall tumour response to definitive chemoradiotherapy, pre‐treatment serum albumin level, post‐treatment serum albumin level and post‐treatment NLR were significantly associated with OS (Table 3). Among them, post‐treatment albumin level, post‐treatment NLR and overall tumour response to definitive chemoradiotherapy remained significant in multivariate analysis (Table 3). The median OS of patients who had SD and PD at post‐treatment evaluation were only 4.4 months (95% CI, 0.9–7.9) and 4.0 months (95% CI, 2.6–5.5), respectively, whereas those of patients who achieved CR and PR were 65.0 months (95% CI, 38.1–92.0) and 17.3 months (95% CI, 12.0‐9–22.5), respectively (Figure 1B).

**TABLE 3:** Univariate and multivariate analysis for overall survival in the entire study population

Variables	Median (95% CI)	Univariate analysis		Multivariate analysis	
		HR (95% CI)	*p* value	HR (95% CI)	*p* value
Age			0.671	Not included	
≥70 years	34.0 (22.3–45.7)	1			
<70 years	18.8 (13.2–24.4)	1.06 (0.82–1.37)			
Sex			0.019		0.095
Female	85.1 (NE)	1		1	
Male	22.2 (16.0–28.4)	2.22 (1.14–4.31)		1.86 (0.90–3.84)	
ECOG PS			0.024		0.305
0	34.4 (4.5–64.3)	1		1	
≥1 or unknown	22.0 (16.1–27.8)	1.45 (1.05–2.01)		1.21 (0.84–1.73)	
Tumour location			0.125	Not included	
Cervical	99.4 (71.9–126.8)	1			
Upper thoracic	18.6 (8.0–29.2)	2.84 (1.19–6.76)			
Mid thoracic	22.2 (9.9–34.5)	2.62 (1.14–6.02)			
Lower thoracic	24.0 (14.4–33.6)	2.50 (1.10–5.67)			
Histologic grade			0.946	Not included	
G1 (W/D)	31.4 (0–62.9)	1			
G2 (M/D)	21.0 (11.5–30.6)	1.05 (0.74–1.49)			
G3 (P/D)	21.9 (9.1–34.7)	0.96 (0.60–1.52)			
GX (not assessed)	27.0 (14.4–39.5)	0.94 (0.54–1.65)			
Clinical TNM stage^a^			0.012		0.324
I	72.6 (56.8–88.4)	1		1	
II	29.3 (20.7–37.9)	1.64 (1.03–2.59)		1.53 (0.93–2.51)	
III	16.5 (9.6–23.4)	1.84 (1.15–2.94)		1.27 (0.75–2.17)	
IV	14.6 (5.4–23.9)	2.19 (1.36–3.53)		1.52 (0.83–2.76)	
Reason for dCRT			0.005		0.622
Patient's refusal	36.2 (18.1–54.2)	1		1	
Medical comorbidity	27.2 (18.2–36.3)	1.32 (0.96–1.82)		1.19 (0.84–1.69)	
Unresectable disease	12.9 (8.5–17.4)	1.61 (1.21–2.15)		1.07 (0.73–1.56)	
Induction chemotherapy			0.438	Not included	
Done	23.9 (16.6–31.3)	1			
Not done	27.2 (0–57.7)	1.20 (0.76–1.90)			
Overall response to dCRT^b^			<0.0001		<0.0001
Complete response	65.0 (38.1–92.0)	1		1	
Partial response	17.3 (12.0–22.5)	2.22 (1.68–2.94)		1.87 (1.37–2.57)	
Stable disease	4.4 (0.9–7.9)	14.4 (6.07–34.4)		10.59 (4.23–26.52)	
Progressive disease	4.0 (2.6–5.5)	12.3 (7.83–19.19)		18.18 (10.40–31.77)	
Weight loss during dCRT			0.001		0.286
<5%	33.9 (18.6–49.1)	1		1	
≥5%	14.0 (9.2–18.9)	1.56 (1.21–2.01)		1.17 (0.88–1.55)	
Pre‐treatment albumin			0.014		0.202
≥3.5 g/dL	27.0 (19.8–34.1)	1		1	
<3.5 g/dL	14.4 (7.6–21.2)	1.47 (1.08–1.99)		1.24 (0.89–1.74)	
Post‐treatment albumin			<0.0001		0.005
≥3.5 g/dL	32.0 (22.2–41.8)	1		1	
<3.5 g/dL	14.0 (9.3–18.8)	1.69 (1.32–2.16)		1.53 (1.14–2.05)	
Pre‐treatment NLR			0.058	Not included	
≤median	29.3 (11.7–47.0)	1			
>median	18.8 (10.0–27.6)	1.28 (0.99–1.64)			
Post‐treatment NLR			0.004		0.004
≤median	33.9 (16.1–51.7)	1		1	
>median	18.3 (10.5–26.1)	1.44 (1.12–1.86)		1.49 (1.14–1.96)	
Pre‐treatment PLR			0.376	Not included	
≤median	29.3 (11.7–47.0)	1			
>median	18.8 (10.0–27.6)	1.12 (0.87–1.43)			
Post‐treatment PLR			0.057	Not included	
≤median	29.3 (20.8–37.9)	1			
>median	18.7 (10.4–26.9)	1.27 (0.99–1.64)			

Abbreviations: CI, confidence interval; dCRT, definitive chemoradiotherapy; ECOG PS, Eastern Cooperative Oncology Group performance status; HR, hazard ratio; M/D, moderately differentiated; NE, not estimated; NLR, neutrophil‐to‐lymphocyte ratio; P/D, poorly differentiated; PLR, platelet‐to‐lymphocyte ratio; W/D, well differentiated.

^a^ Clinical staging was done according to the eighth edition of the American Joint Committee on Cancer staging system.

^b^ Overall response was classified mainly based on EORTC‐PET criteria^12^ with the results of endoscopy and computed tomography.

**FIGURE 2:**
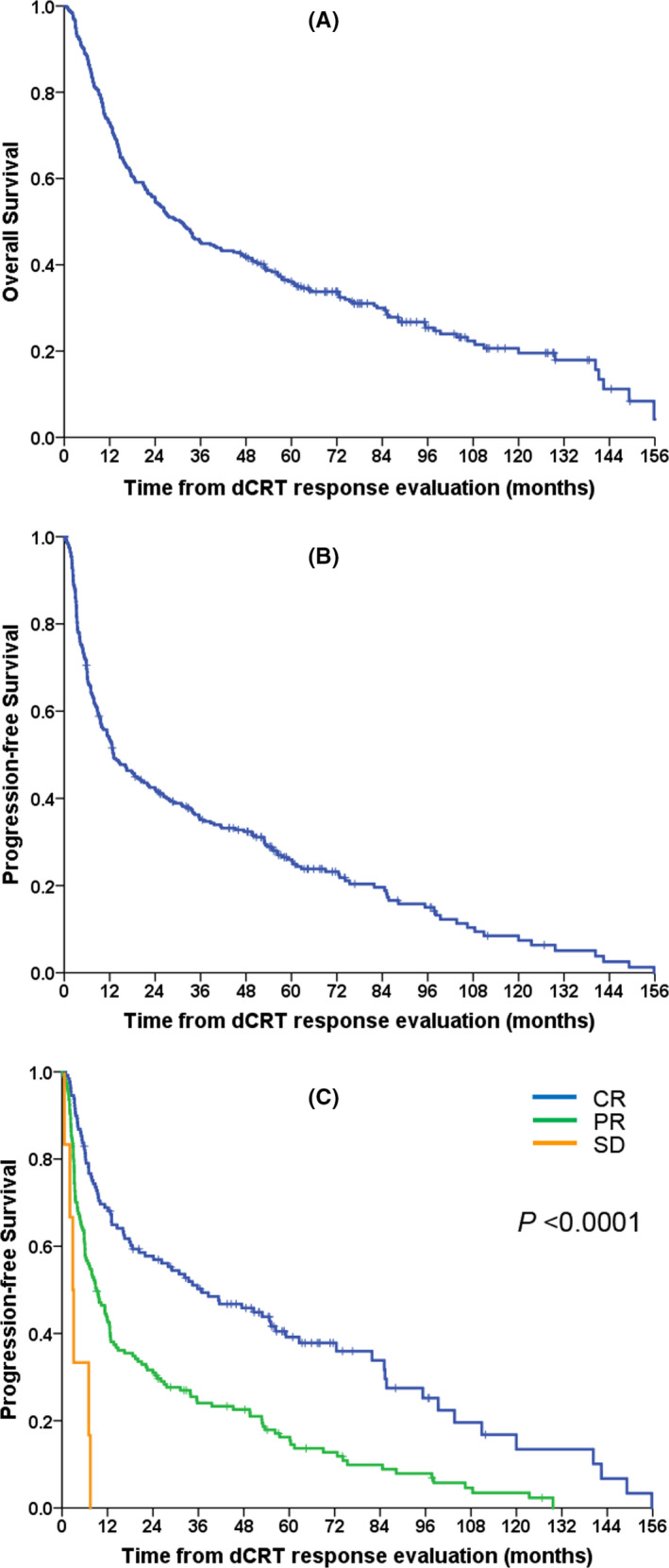
(A) Overall survival curve and (B) progression‐free survival curve of patients who achieved clinical stabilisation including complete response (CR), partial response (PR) and stable disease (SD) at the end of definitive chemoradiotherapy (dCRT) and (C) progression‐free survival curves according to overall tumour response to definitive chemoradiotherapy (dCRT)

Among patients who showed disease stabilisation including CR, PR or SD at the evaluation of the response to definitive chemoradiotherapy, the median OS and PFS from post‐treatment response evaluation were 30.5 months (95% CI, 23.6–37.3) and 13.1 months (95% CI, 8.9–17.3), respectively (Figure 2A, B). The OS rates at 1, 3 and 5 years from post‐treatment response evaluation were 73.0% (95% CI, 67.9–78.0%), 45.3% (95% CI, 39.6–50.9) and 36.1% (95% CI, 30.6–41.5), respectively, and the PFS rates at 1, 3 and 5 years from post‐treatment response evaluation were 53.6% (95% CI, 47.9–59.4%), 35.1% (95% CI, 29.5–40.6%) and 26.0% (95% CI, 20.6–31.3%), respectively. In prognostic factor analysis for the OS and PFS of these patients, significant factors in univariate analysis were sex, ECOG performance status, overall tumour response to definitive chemoradiotherapy, weight loss during definitive chemoradiotherapy, pre‐treatment albumin level, post‐treatment albumin level, pre‐treatment NLR and post‐treatment NLR for OS, and sex, ECOG performance status, cTNM stage, overall clinical response to definitive chemoradiotherapy, weight loss during definitive chemoradiotherapy, post‐treatment albumin level, post‐treatment NLR and post‐treatment PLR for PFS (Table 4). However, only overall tumour response to definitive chemoradiotherapy remained significant in multivariate analysis for both PFS and OS (Table 4). Patients who showed clinical CR had excellent survival, with a median OS rate of 65.0 months and a median PFS of 36.9 months from the date of response evaluation; however, compared to them, patients who showed PR had a 1.84 times higher risk of progression (median PFS, 9.2 months [95% CI, 6.1–12.2]) and a 2.18 times higher risk of death (median OS, 17.3 months [95% CI, 12.0–22.5]), and those with SD had a 6.44 times higher risk of progression (median PFS, 2.8 months [95% CI, 1.6–4.0]) and a 13.43 times higher risk of death (median OS, 4.4 months [95% CI, 0.9–7.9]) (Figures 1B, 2C, Table 3).

**TABLE 4:** Univariate and multivariate analysis for the OS and PFS of patients who achieved disease stabilisation after dCRT.

Variables	OS					PFS				
	Median (95% CI)	Univariate analysis		Multivariate analysis		Median (95% CI)	Univariate analysis		Multivariate analysis	
		HR (95% CI)	*p* value	HR (95% CI)	*p* value		HR (95% CI)	*p* value	HR (95% CI)	*p* value
Age			0.429	—				0.972		
≥70 years	34.4 (22.7–46.1)	1				24.1 (9.2–39.1)	1			
<70 years	26.3 (17.8–34.9)	0.90 (0.69–1.17)				12.2 (8.3–16.0)	0.99 (0.77–1.29)			
Sex			0.040		0.107			0.028		0.068
Female	58.1 (NE)	1		1		85.1 (22.2–147.9)	1		1	
Male	27.6 (20.7–34.5)	2.01 (1.03–3.93)		1.80 (0.88–3.68)		12.8 (9.0–16.7)	2.12 (1.09–4.12)		1.97 (0.95–4.05)	
ECOG PS			0.030		0.106			0.046		0.142
0	52.8 (29.6–76.0)	1		1		28.0 (6.7–49.4)	1		1	
≥1 or unknown	26.3 (19.2–33.5)	1.47 (1.04–2.07)		1.36 (0.94–1.96)		12.6 (8.3–16.8)	1.39 (1.01–1.93)		1.01 (0.92–1.87)	
Tumour location			0.163	—				0.153		
Cervical	99.4 (71.9–126.8)	1				99.4 (0–214.9)	1			
Upper thoracic	21.4 (12.5–30.2)	2.74 (1.14–6.57)	0.024			7.4 (0–16.5)	2.52 (1.11–5.71)	0.027		
Mid thoracic	31.7 (14.9–48.5)	2.38 (1.03–5.48)	0.042			12.9 (8.2–17.5)	1.93 (0.89–4.21)	0.097		
Lower thoracic	30.5 (22.5–38.5)	2.30 (1.01–5.25)	0.047			14.3 (6.6–21.9)	1.92 (0.89–4.14)	0.095		
Histologic grade			0.934	—				0.845		
G1 (W/D)	36.2 (4.75–67.6)	1				12.5 (0–31.5)	1			
G2 (M/D)	27.3 (17.2–37.2)	1.06 (0.73–1.53)				12.8 (7.3–18.4)	1.15 (0.80–1.66)	0.447		
G3 (P/D)	24.7 (13.2–36.3)	0.96 (0.58–1.57)				13.7 (7.0–20.3)	1.09 (0.67–1.78)	0.736		
GX	28.8 (3.8–53.8)	0.94 (0.52–1.70)				24.0 (4.5–43.4)	0.99 (0.55–1.79)	0.978		
Clinical TNM stage^a^			0.122					0.023		0.421
I	72.6 (56.8–88.4)	1				52.8 (29.6–76.0)	1		1	
II	32.4 (23.6–41.3)	1.58 (1.00–2.50)	0.052			21.0 (9.9–32.2)	1.49 (0.96–2.31)	0.078	1.20 (0.74–1.93)	0.458
III	18.8 (8.7–28.8)	1.67 (1.04–2.70)	0.035			10.3 (5.6–15.1)	1.64 (1.04–2.59)	0.035	1.21 (0.73–2.00)	0.456
IV	22.0 (6.2–37.7)	1.80 (1.09–2.95)	0.021			8.7 (5.2–12.2)	2.05 (1.28–3.27)	0.003	1.52 (0.90–2.58)	0.120
Reason for dCRT			0.081					0.118		
Patient's refusal	41.5 (24.8–58.3)	1				21.9 (12.0–31.8)	1			
Comorbidity	27.6 (17.1–38.2)	1.34 (0.96–1.86)				14.3 (3.0–25.5)	1.20 (0.87–1.66)	0.258		
Unresectable	17.5 (10.3–24.6)	1.38 (1.01–1.89)				6.4 (4.0–8.8)	1.38 (1.01–1.87)	0.040		
Induction chemotherapy			0.264					0.150		
Done	31.0 (24.2–37.9)	1				13.1 (8.3–17.8)	1			
Not done	27.2 (0–56.3)	1.32 (0.81–2.14)				12.6 (10.3–14.9)	1.42 (0.88–2.27)			
Overall response to dCRT^b^			<0.0001		<0.0001			<0.0001		<0.0001
CR	65.0 (38.1–92.0)	1		1		36.9 (17.9–56.0)	1		1	
PR	17.3 (12.0–22.5)	2.24 (1.70–2.96)		2.18 (1.59–2.98)		9.2 (6.1–12.2)	2.10 (1.60–2.75)	<0.0001	1.84 (1.37–2.47)	<0.0001
SD	4.4 (0.9–7.9)	15.9 (6.59–38.15)		13.43 (5.25–34.35)		2.8 (1.6–4.0)	7.77 (3.33–18.10)	<0.0001	6.44 (2.64–15.71)	<0.0001
Weight loss during treatment			0.004		0.336			0.025		0.555
<5%	38.6 (22.4–54.8)	1		1		21.9 (12.6–31.3)	1		1	
≥5%	20.8 (12.2–29.4)	1.48 (1.13–1.94)		1.16 (0.86–1.57)		9.3 (5.7–12.9)	1.35 (1.04–1.76)		1.09 (0.81–1.47)	
Pre‐treatment albumin			0.025		0.305			0.071		
≥3.5 g/dL	33.4 (21.2–45.7)	1		1		16.3 (10.1–22.4)	1			
<3.5 g/dL	17.3 (8.0–26.6)	1.45 (1.05–2.01)		1.21 (0.84–1.73)		7.5 (1.2–13.7)	1.35 (0.98–1.85)			
Post‐treatment albumin			<0.0001		0.131			0.015		0.220
≥3.5 g/dL	36.2 (19.0–53.3)	1		1		18.7 (10.1–27.3)	1		1	
<3.5 g/dL	17.1 (8.6–25.5)	1.70 (1.30–2.22)		1.27 (0.93–1.73)		9.2 (6.7–11.6)	1.38 (1.07–1.80)		1.21 (0.89–1.65)	
Pre‐treatment NLR			0.047		0.656			0.064		
≤median	44.7 (24.6–64.8)	1		1		19.4 (9.4–29.3)	1			
>median	24.7 (13.8–35.6)	1.31 (1.00–1.72)		0.93 (0.69–1.27)		11.3 (8.6–14.0)	1.28 (0.99–1.66)			
Post‐treatment NLR			0.010		0.073			0.019		0.317
≤median	44.7 (27.7–61.8)	1		1		24.1 (8.7–39.6)	1		1	
>median	23.9 (16.3–31.6)	1.43 (1.09–1.87)		1.31 (0.98–1.76)		9.7 (5.7–13.6)	1.37 (1.05–1.78)		1.18 (0.86–1.61)	
Pre‐treatment PLR			0.504					0.157		
≤median	33.9 (18.6–49.1)	1				14.8 (7.0–22.5)	1			
>median	24.7 (13.2–36.3)	1.09 (0.84–1.43)				12.5 (6.3–18.8)	1.20 (0.93–1.55)			
Post‐treatment PLR			0.081					0.015		0.147
≤median	36.2 (23.4–49.0)	1				24.0 (9.6–38.3)	1		1	
>median	21.0 (13.3–28.7)	1.27 (0.97–1.65)				8.8 (4.3–13.4)	1.38 (1.06–1.78)		1.26 (0.92–1.72)	

Abbreviations: CI, confidence interval; CR, complete response; dCRT, definitive chemoradiotherapy; ECOG PS, Eastern Cooperative Oncology Group performance status; GX, grade not assessed; HR, hazard ratio; M/D, moderately differentiated; NE, not estimated; NLR, neutrophil‐to‐lymphocyte ratio; OS, overall survival; P/D, poorly differentiated; PFS, progression‐free survival; PLR, platelet‐to‐lymphocyte ratio; PR, partial response; SD, stable disease; W/D, well differentiated.

^a^ Clinical staging was done according to the eighth edition of the American Joint Committee on Cancer staging system.

^b^ Overall clinical response was classified mainly based on EORTC‐PET criteria^12^ with the results of endoscopy and computed tomography.

### 3.4 Pattern of first progression or recurrence according to the response to definitive chemoradiotherapy

At the time of initial response evaluation to definitive chemoradiotherapy, 31 patients (9.5%) had PD. Among the remaining 296 patients who had non‐PD response at the time of first post‐treatment evaluation, 152 (51.4%) had disease progression or recurrence at follow‐up. The pattern of the first failure was significantly different between the PD group and non‐PD group. In the initial PD group of patients, distant metastases were predominant (87.1% experienced distant metastases, 54.8% experienced locoregional progression). On the contrary, in patients who showed initial non‐PD response but experienced disease progression at subsequent follow‐up, the predominant pattern of failure was locoregional recurrence/progression (81.6% experienced locoregional recurrence/progression, 39.5% experienced distant metastases) (*p* < 0.0001) (Table 5).

**TABLE 5:** Pattern of first progression or recurrence

Overall clinical response at the end of dCRT^b^	PD/recurrence	Pattern of disease progression or recurrence			*p* value^a^
		Locoregional lesion only	Distant lesion only	Both	
PD (*n* = 31)	31 (100%)	4 (12.9%)	14 (45.2%)	13 (41.9%)	<0.0001
non‐PD (*n* = 296)	152 (51.4%)	92 (60.5%)	28 (18.4%)	32 (21.1%)	
CR (*n* = 132)	53 (40.2%)	35 (66.0%)	9 (17.0%)	9 (17.0%)	
PR (*n* = 158)	95 (60.1%)	54 (56.8%)	18 (18.9%)	23 (24.2%)	
SD (*n* = 6)	4 (66.7%)	3 (75.0%)	1 (25.0%)	0 (0%)	

Abbreviations: CR, complete response; dCRT, definitive chemoradiotherapy; PD, progressive disease; PR, partial response; SD, stable disease.

^a^ Overall response was classified mainly based on EORTC‐PET criteria^12^ with the results of endoscopy and computed tomography.

^b^ *p* value for the PD group versus the non‐PD group.

## 4 DISCUSSION

In this study, we evaluated the clinical outcome and prognostic factors in definitive chemoradiotherapy‐treated localised oesophageal cancer patients, and demonstrated that although 91% of the patients initially achieve disease stabilisation, 51% of them experience recurrence. However, the survival outcome differed significantly according to the clinical response after definitive chemoradiotherapy, with hazard ratios of PR and SD response of 2.2 and 13.4 for OS, and 1.8 and 6.4 for PFS, when compared with CR response.

Although the standard of care for patients with locally advanced oesophageal cancer includes surgical resection, definitive chemoradiotherapy is also considered as an alternative to neoadjuvant chemoradiotherapy followed by surgery. In clinical practice, a considerable number of patients with resectable locally advanced oesophageal cancer refuse surgery mainly due to potential morbidity and mortality following oesophagectomy and instead receive definitive chemoradiotherapy.^14^ Furthermore, definitive chemoradiotherapy is the preferred treatment option for cervical or cT4b oesophageal cancer. However, the prognosis of patients treated with definitive chemoradiotherapy has not been satisfactory. Previous phase III studies and large sized retrospective studies showed median OS of 13–18 months and a 3‐year OS rate of 19–26% in localised oesophageal cancer treated with definitive chemoradiotherapy.^6^, ^15^, ^16^

Despite these unsatisfactory outcomes, the current common clinical practice is surveillance without further treatment until disease progression or recurrence in patients who showed response or stabilisation after chemoradiotherapy, unless salvage surgery is indicated. Previous retrospective studies have failed to show the benefits of consolidation or adjuvant chemotherapy after definitive chemoradiotherapy for oesophageal cancer.^17^, ^18^ However, as patient populations receiving definitive chemoradiotherapy are heterogeneous in terms of the reason for definitive chemoradiotherapy, disease severity, response to definitive chemoradiotherapy, etc., their prognosis could be different depending on specific prognostic factors. The identification of these prognostic factors could help establish treatment strategies after definitive chemoradiotherapy in clinical practice or clinical trials.

Unlike previous studies, we evaluated OS and PFS from the date of response evaluation for definitive chemoradiotherapy, and not the date of the start of treatment, to investigate the prognosis and associated prognostic factors after definitive chemoradiotherapy. Besides, we evaluated treatment response by combining multiple clinical evaluation modalities including EGD and CT as well as FDG‐PET. This comprehensive assessment could improve the accuracy in determining tumour response compared to PET alone which could not exclude the presence of microscopic or macroscopic residual cancer in cases with complete metabolic response or diffuse oesophagitis,^19^ and could not play a role in cases with non‐FDG‐avid tumours.

We found that post‐treatment albumin level, post‐treatment NLR and overall tumour response were independent prognostic factors in patients who received definitive chemoradiotherapy. A high NLR, a potential marker of systemic inflammation, has been reportedly associated with reduced treatment response and worse outcomes in many solid tumours, including oesophageal cancer.^20^, ^21^, ^22^ Serum albumin is an objective parameter that is closely correlated with the degree of malnutrition, and a decrease in serum albumin level during or after definitive chemoradiotherapy has been reported as a poor prognostic factor in oesophageal cancer patients.^23^, ^24^ However, the most powerful prognostic factor in the present study was overall tumour response to definitive chemoradiotherapy. We categorised responses to chemoradiotherapy into four subgroups (CR, PR, SD and PD), rather than dividing them into dichotomous groups (CR and non‐CR), and found that prognosis after chemoradiotherapy including OS and PFS was markedly different according to this detailed response classification. Patients who achieved CR had excellent survival (median OS, 65.0 months), and those who achieved PR showed intermediate prognosis (median OS, 17.3 months); in contrast, patients who failed to achieve clinical response had very poor survival (median OS, 4.4 months for SD, 4.0 months for PD) (Figure 1B). This markedly different prognosis after definitive chemoradiotherapy suggests the need for different treatment strategies according to clinical responses. For example, it would be reasonable to closely follow‐up without further treatment for patients who achieved CR, whereas additional consolidation treatment strategy should be developed for patients who had PR. For patients who showed SD, more aggressive salvage therapy should be considered given they had very poor prognosis similar to patients with PD. Currently, several clinical trials for consolidation therapy after definitive chemotherapy using immune checkpoint inhibitors are ongoing in oesophageal cancers, but most of them did not include treatment response after chemoradiotherapy as a stratification factor.^25^, ^26^, ^27^, ^28^, ^29^, ^30^

Interestingly, the pattern of failure after definitive chemoradiotherapy was also significantly different according to the clinical response. De novo progression to definitive chemoradiotherapy was manifested as both locoregional (54.8%) and distant (81.5%) failures, suggesting insensitivity to both radiotherapy and chemotherapy in these patients. In contrast, the predominant locoregional failure pattern in patients who showed CR, PR or SD in response to chemoradiotherapy indicated the importance of surveillance after definitive chemoradiotherapy considering the possibility of salvage surgery for progression in these patients. Further studies are warranted to establish an optimal surveillance strategy after definitive chemoradiotherapy.

There are limitations in our study. First, our study included a heterogeneous group of patients in terms of clinical stage and the reason for definitive chemoradiotherapy. However, multivariate analyses presented in this study included these variables for adjustment. Second, we evaluated treatment response 4–8 weeks after the end of chemoradiotherapy when inflammatory processes could be still ongoing in a considerable proportion of patients. Therefore, the diagnostic yield for FDG‐PET in differentiating radiation oesophagitis from residual disease might be limited. However, we attempted to minimise this issue by combining EGD (with biopsy), CT and FDG‐PEG as response assessment modalities, and showed that the response evaluation using this combined clinical assessment was able to predict the prognosis after definitive chemoradiotherapy. Despite these limitations, our study provides useful information on the prognostic value of comprehensive overall response evaluation after definitive chemoradiotherapy.

## 5 CONCLUSIONS

In conclusion, patients receiving definitive chemoradiotherapy for oesophageal squamous cell carcinoma make up a heterogeneous population showing highly different prognoses after treatment, especially according to the clinical response to chemoradiotherapy. Further studies are required on the role of additional treatment and individualised treatment strategy according to the response to treatment.

## 6 CONFLICT OF INTEREST STATEMENT

The authors declare no competing interests.

## Supporting information

Table S1

## Data Availability

The data sets generated and analysed during the current study are available from the corresponding author on reasonable request.
