# Supplementary material for: Analysis of clinical outcomes and prognostic factors in patients treated with definitive chemoradiotherapy for oesophageal squamous cell carcinoma
Source: Cancer Med. 2021 Feb 15;10(5):1745–58. doi: 10.1002/cam4.3783 (PMC7940212; doi:10.1002/cam4.3783)
Supplement: Supplementary file 1 — Table S1 [file CAM4-10-1745-s001.docx]

**Supplementary table S1. Summarization of overall and metabolic tumour response**

| Metabolic response | Overall response* | | | |
| --- | --- | --- | --- | --- |
|  | **CR** | **PR** | **SD** | **PD** |
| CMR | 123 (93.2%) | 23 (14.6%) | 0 (0.0%) | 0 (0.0%) |
| PMR | 0 (0.0%) | 115 (72.8%) | 0 (0.0%) | 3 (9.7%) |
| SMD | 0 (0.0%) | 0 (0.0%) | 6 (100.0%) | 1 (3.2%) |
| PMD | 0 (0.0%) | 0 (0.0%) | 0 (0.0%) | 27 (87.1%) |
| Diffuse esophagitis | 7 (5.3%) | 19 (12.0%) | 0 (0.0%) | 0 (0.0%) |
| Non-FDG avid tumour | 2 (1.5%) | 1 (0.6%) | 0 (0.0%) | 0 (0.0%) |

*Overall response was classified mainly based on EORTC-PET criteria^12^ with the results of endoscopy and computed tomography (described in the Methods section in detail).

CR, complete response; PR, partial response; SD, stable disease; PD, progressive disease; CMR, complete metabolic response; PMR, partial metabolic response; SMD, stable metabolic disease; PMD, progressive metabolic disease; FDG, ^18^F-fluorodeoxyglucose.
